# Hypotheses concerning how Otx2 makes its incredible journey: a hitchhiker on the road to Rome?

**DOI:** 10.3389/fnmol.2013.00055

**Published:** 2013-12-30

**Authors:** Lawrence Edelstein, John Smythies

**Affiliations:** ^1^Medimark CorporationDel Mar, CA, USA; ^2^Integrative Neuroscience Program, Department of Psychology, Center for Brain and Cognition, University of California San DiegoLa Jolla, San Diego, CA, USA

**Keywords:** homeoproteins, Otx2, axonal transport, trans-synaptic, exosomes, PVs, epigenetics

## Introduction

From work carried out largely by Alain Prochiantz and his co-workers at the College de France (Sugiyama et al., 2008; Beurdeley et al., 2012; Carlier et al., 2013; Smythies and Edelstein, 2013; Spatazza et al., 2013a,b), it is now known that the homeoprotein and transcription factor molecule Otx2 possesses a very unusual property. This flexible molecule is able to cross cell membranes without using a specific energy-requiring carrier. The entry mechanism involves organizing a micelle structure out of the planar lipoprotein plasma membrane via interactions between charged residues and lipophilic interactions. This micelle carries the molecule of Otx2 across the membrane. It has been shown that the membrane exit mechanism of a related molecule, Engrailed 2, is different and involves the formation of energy-dependent calveoli-like vesicles (Sugiyama et al., 2008; Carlier et al., 2013; Spatazza et al., 2013a). These vesicles are delivered into the interior of the cell.

When injected into the eye Otx2 is transported to the visual cortex where it enters PV-expressing GABAergic INs (PVs), which it activates (Sugiyama et al., 2008; Beurdeley et al., 2012; Carlier et al., 2013; Spatazza et al., 2013a,b). During natural development, following the opening of the eyes, Otx2 molecules accumulate “gradually” in the visual cortex. *In vivo*, the molecule at source is synthesized in a retinal bipolar cell and transmitted to a retinal ganglion cell. The ganglion cell contains no Otx2 mRNA. At the other end of the journey, neither does the cortical cell; its Otx2 is transmitted from neurons in the lateral geniculate nucleus (LGN).

## Axonal transport

Molecules are usually carried along axons by one of two mechanisms (Kaether et al., 2000; Roy, 2013). Individual proteins, bundled into packets called multiprotein complexes, are carried by the slow system. This moves at a speed of less than 0.8 cm a day. Vesicles, on the other hand, are carried by the fast system at speeds of up to 20 cm a day. The distance between the retina and the visual cortex in mice is about 1 cm. Thus a molecule taking the fast route could make the journey in about 1 h, whereas the minimum time for a molecule taking the slow route would be about 24 h—not allowing for the time needed to cross two synapses. The time that an Otx2 molecule takes to get from the retina to the cortex is 24 h (Prochiantz, personal communication). The rate of build-up would also depend on the amount of the molecule bring transmitted and the rate of diffusion at the target. Presumably, Otx2 would use one of these axonal transport mechanisms during its travel along axons, as it is difficult to visualize it wriggling on its own along axons from one cell to the next for these long distances.

## The rest of the journey

Here we present three hypotheses (that are not necessarily mutually exclusive—that is to say the molecule of Otx2 could use different mechanisms for different parts of its journey).

### Hypothesis 1

It could cross each intervening membrane on its own without any carrier mechanism, by the molecular mechanism as described above. It could also cross the synaptic cleft by itself via the cytoplasm, nanotubules Marzo et al., 2012; Fuxe et al., 2013; Kinney et al., 2013, or via perisynaptic astrocytes (Smythies and Edelstein, 2013). Sugiyama et al. (2008) have shown that it can cross the plasma membrane of the target PVs, since cortical infusion over 5 days results in robust Otx2 signaling throughout the visual cortex. But how does the molecule of Otx2 avoid false trails on its sojourn? This may be answered by what we call an “All roads lead to Rome” hypothesis. Since the Otx2 molecule is carrying no other information other than that the eye is being stimulated, then a diffuse projection might prove sufficient. The Otx2 molecule could take any path from the retina to the cortex, so long as it was along a visual axon. The diffuse scattering of Otx2 molecules in the LGN reported by Sugiyama et al. (2008) may support this. These authors say “In the LGN, Otx2 protein was diffusely spread over multiple cells unlike Otx2 transcripts found within discrete GABAergic interneurons.” Another objection is that this model would seem to need an inordinate number of specific cell recognition lipo/sugar sites on the surface of all the membranes it has to cross en route to its target. Furthermore, these recognition sites are only located on the surface of PV neurons (Beurdeley et al., 2012; Spatazza et al., 2013a,b). However, if it is fed into a transport system that is more or less sealed from its surroundings (i.e., the optic nerve and optic radiation) where there is a great deal of anterograde traffic, perhaps it is swept along by the flood?

### Hypothesis 2

Otx2 could use a mechanism similar to that employed by botulinum toxin (Akaike et al., 2013). Botulinum neurotoxins (NTXs) are transported both antero- and retrogradely along either motor or sensory axons for bidirectional delivery in the central nervous system. This mode of travel involves the recycling of synaptic vesicles. A newly developed type of NTX, A2 NTX (A2NTX), injected into one rat foreleg muscle, was transported to the contralateral muscle. This finding indicates that the NTX traveled retrogradely via spinal neurons and then transsynaptically through motor neurons to the contralateral motor neurons within the spinal cord, and then anterogradely to the soleus muscle (Akaike et al., 2013). Following BoNT-A injection into the rat eye, significant levels of BoNT-A-cleaved SNAP-25 appeared in the superior colliculus (Restani et al., 2011).

### Hypothesis 3

Otx2 could use the exosome system in a variety of ways. Exosomes are lipoprotein vesicles given off by nearly all cells and are used to exchange epigenetic material, such as protein transcription factors and a variety of RNAs (Smythies and Edelstein, 2013). They are constructed inside multivesicular bodies (MVBs) located, in the case of neurons, at the axon terminal of a pre-synaptic neuron A. Here, a complex mechanism in which RNA-induced silencing complexes (RISC) may be involved (Pegtel et al., 2011, p. 15), loads them with very specific mixtures of epigenetic molecules. The loaded exosomes are then transported to the plasma membrane, which they cross, and are further transported across the synapse, most likely via astrocytes and nanotubules (Marzo et al., 2012; Fuxe et al., 2013; Kinney et al., 2013), to be delivered to the interior of the post-synaptic neuron B. At this point, some release their cargoes locally, while others are transported to the perinuclear zone where they discharge their payloads in order to modulate transcription (Smythies and Edelstein, 2013). We will discuss what may happen to these latter vesicles themselves later.

In this account, however, we have noticed that something appears to be missing. Where do the proteins and nucleic acids that are loaded into the exosome come from? We have not yet seen this specific question addressed in the literature. The state of our knowledge on this subject was put recently by Vlassov et al. (2012) as “The packaging of specific cargo loads with appropriate destination tags itself is extremely intriguing and currently a black box ….” This process is modulated by heparanase (Thompson et al., 2013) and tetraspanins (Perez-Hernandez et al., 2013). As the ingredients of the cargo presumably do not float in from the cytoplasm, there seems to be only one possible source—synthesis by the nucleus of the pre-synaptic neuron. This would involve a carrier mechanism to transport them from the perinuclear zone to the axon terminal. Presumably, this would consist of specific vesicles, which we can call pre-exosomes, carried by the well-established dynein-based fast axonal transport mechanism.

This raises a rather interesting second question. Where do these pre-exosomes come from? The perinuclear zone exports many differently-specified vesicles to carry the many different proteins down the axon. Are pre-exosomes derived from this general pool? Conceivably, however, there may be another source. As we have seen above, the perinuclear zone of a post-synaptic neuron B transsynaptically receives exosomes carrying epigenetic cargoes that originated from the pre-synaptic neuron A. Therefore, it seems possible that these exosomes, after being emptied of their cargo, could be recycled and used as pre-exosomes to transport new protein and nucleic acid epigenetic payloads from the nucleus of post-synaptic neuron B to its own exosome factory in its own axon terminal that forms a synapse with post-synaptic neuron C, one neuron further along the network. Such recycling of vesicles and their membranes is found elsewhere in the brain—for example, in the case of synaptic vesicles where a single vesicle may be used hundreds of times. In the transsynaptic part of the exosome system itself, exosomes are ferried in both directions across the synapse (Smalheiser, 2007). Thus, exosomes may not only be exporting protein transcription factors and nucleic acids, but quantities of membrane as well. This membrane material could pass down a chain of synaptically-connected neurons potentially carrying information. We can use this mechanism to construct an hypothesis to explain how the unusual transcription protein Otx2 could act.

Our third hypothesis (of those listed above) suggests the following mechanism. The molecule of Otx2 is synthesized in the bipolar cell in the retina and crosses into the ganglion cell by the direct transmembrane mechanism described above. It then enters the cytoplasm of the ganglion cell inside its calveoli-like vesicle. This vesicle may then either be directly loaded onto the fast transport system, or fused with an already existing pre-load-bearing exosome, to be pulled by the dynein motor along the microtubule track. The contained Otx2 molecule could also be bundled into a multiprotein complex and carried by the slow route. Since Otx2 is an epigenetic transcription protein, and that the pre-exosome is specialized for the transport of epigenetic molecules, the Otx2 molecule may possess the right molecular identification codes to effect this transmission. This now puts the Otx2 molecule inside the exosome system as cargo within a pre-exosome. The pre-exosome may then be carried down the ganglion cell axon along the optic nerve to the MVBs in the axon terminal of the synapse with an LGN neuron. Here, it is processed by the mechanism described above, in which its cargo is delivered, shuffled and reloaded into exosomes. These cross the synapse and are further transported to the perinuclear zone of the LGN neuron. They then release their payloads and the Otx2 molecules are taken-up by the second fast axonal transport mechanism, where they are sent on their way to the next synapse in the chain that the LGN axon makes with the PV cell. The exosome then releases its cargo into the synaptic cleft to allow the Otx2 molecules to bind to the polysaccharide receptors on the PNNs of the PV target neuron in the manner described by Sugiyama et al. (2008), Beurdeley et al. (2012) and Spatazza et al. (2013a,b). However, against this hypothesis is the fact that no homeoproteins have so far been reported as constituents of exosome payloads. A place to look for them might be the pathway from retina to visual cortex. It is quite possible that the calveoli-like vesicles described above, in which Otx2 molecules are delivered into the lumen of the axon, are treated as exosomes from that point on but are not identified as such by current tests and methodologies.

Continuous accumulation of Otx2 in PV cells may be necessary to sustain a state that limits further plasticity in the adult (Beurdeley et al., 2012). When this supply is cut off by deafferentation, and replaced by an input belonging to another sensory modality, this may allow the cell to undergo plastic changes organized perhaps by a new modality specific homeoprotein carried by the new input, and by epigenetic material carried by exosomes (Smythies and Edelstein, 2013). However, Sugiyama et al. (2008) have shown that Otx2 suffused into an immature visual cortex is sufficient to promote the full growth and functionality of PV neurons. Therefore, the full contribution of exosome payloads to the epigenetics of the post-synaptic neuron must be something other than acting on PV cells in this way. Epigenetic cortical restructuring involves many neuronal locations and species other than PV cells, such as dendrites and spines (Smythies and Edelstein, 2013). Perhaps the microRNAs and other chemical constituents transferred by exosomes play a role in these events. It seems reasonable to suggest that the abundant and diverse cargo of epigenetic material carried by exosomes must have some epigenetic function in the receiving cell. Hopefully, experiments can be devised to test these hypotheses.
